# Anticoagulation Options for Cranial Procedures: A Comparative Review of Aspirin, Plavix, and Aggrastat

**DOI:** 10.7759/cureus.43899

**Published:** 2023-08-22

**Authors:** Harendra Kumar, Aishwarya Boini, Mpuekela Tshibangu, Bikona Ghosh, Fatima Shaheen, Andrew M Joseph, Juliana Cazzaniga, Monica Karas, Cesar E Jara Silva, Jonathan Quinonez, Samir Ruxmohan

**Affiliations:** 1 Medicine and Surgery, Dow University of Health Sciences, Karachi, PAK; 2 Medicine, Government Medical College and Hospital, Siddipet, IND; 3 Medicine, Davao Medical School Foundation, Davao, PHL; 4 Neurology, Ross University School of Medicine, Bridgetown, BRB; 5 Medicine and Surgery, Dhaka Medical College, Dhaka, BGD; 6 Medicine, M. N. Raju (MNR) Medical College, Hyderabad, IND; 7 Osteopathic Medicine, Nova Southeastern University Dr. Kiran C. Patel College of Osteopathic Medicine, Davie, USA; 8 Herbert Wertheim College of Medicine, Florida International University, Herbert Wertheim College of Medicine, Miami, USA; 9 Osteopathic Medicine, Nova Southeastern University Dr. Kiran C. Patel College of Osteopathic Medicine, Fort Lauderdale, USA; 10 Neurology/Osteopathic Neuromuscular Medicine, Larkin Community Hospital, Miami, USA; 11 Division of Neurocritical Care, University of Texas (UT) Southwestern Medical Center, Dallas, USA

**Keywords:** aggrastat, cranial surgery, aspirin, plavix, intraoperative/postoperative anticoagulation

## Abstract

Anticoagulation therapy is critical to avoiding thrombotic events in patients following cranial surgery. Although Aspirin, Plavix, and Aggrastat are used as anticoagulants for this purpose, there is no consensus on which agent is the most effective and safe. In this comparative study, we analyze the current evidence on the efficacy and safety of these three anticoagulants in the context of cranial surgeries. This review focuses on the advantages and disadvantages of each anticoagulant, such as its pharmacokinetics, indications, contraindications, and possible consequences. The outcomes of this study will help physicians choose the best anticoagulant for their patients based on individual patient characteristics and the kind of cranial procedure. Aggrastat's potential to be included as a recommended anticoagulant for cranial procedures warrants further study.

## Introduction and background

Aspirin, Plavix, and Aggrastat are commonly prescribed antiplatelet agents used to manage various cardiovascular and cerebrovascular diseases. These medications have different mechanisms of action, pharmacokinetics, and pharmacodynamics, which affect their efficacy and safety profiles [1]. Understanding these differences is critical for clinicians to select the most appropriate therapy for their patients and optimize treatment outcomes.

Aspirin, a nonsteroidal anti-inflammatory drug (NSAID), irreversibly inhibits the cyclooxygenase (COX) enzyme and reduces the production of thromboxane A2 (TXA2), a potent platelet activator [2]. Plavix, a thienopyridine derivative, selectively inhibits the adenosine diphosphate (ADP) receptor on platelets and reduces platelet activation and aggregation [3]. Aggrastat, a glycoprotein IIb/IIIa receptor antagonist, blocks the final common pathway of platelet aggregation by preventing fibrinogen from binding to platelet receptors [4].

In addition to their different mechanisms of action, these drugs have distinct pharmacokinetic and pharmacodynamic profiles. Aspirin is rapidly absorbed in the upper gastrointestinal (GI) tract, metabolized by the liver, and excreted by the kidneys [4,5]. Its antiplatelet effect lasts for the lifespan of the platelet, which is about 7-10 days [4,5]. Plavix is a prodrug that requires hepatic activation, and its antiplatelet effect lasts up to 5 days [4,5]. Aggrastat has a short half-life of two to four hours but has a metabolism that is overall limited despite being primarily eliminated by the kidneys [5].

This paper aims to provide a comprehensive overview of the mechanisms of action, pharmacokinetics, and pharmacodynamics of aspirin, Plavix, and Aggrastat. We will discuss the clinical implications of these differences regarding efficacy, safety, and dosing considerations. We will also explore the current evidence for combination therapy with these agents and their role in cranial procedures. The ultimate goal of this paper is to provide clinicians with a better understanding of these commonly used antiplatelet agents and facilitate their decision-making in clinical practice.

## Review

Aspirin, a member of the salicylate family, is an NSAID prototype. Its active agent is salicylic acid [6]. Aspirin's antithrombotic action is due to platelet inhibition by acetylation of platelet COX, resulting in irreversible inhibition of thromboxane formation. Vascular endothelial cells express COX-1 and COX-2, but mature platelets only express COX-1 and produce TXA2. The requirements for aspirin dosage are different as an antithrombotic (COX-1) and an anti-inflammatory drug (COX-2). Low-dose aspirin selectively inhibits COX-1, whereas higher doses inhibit COX-1 and COX-2 [7,8].

Plavix (Clopidogrel) is a prodrug of the thienopyridine family. The metabolism of the prodrug produces the active metabolites (Act-Met). After oral administration, approximately 50% of the dose is absorbed by the intestine. Active metabolites inhibit the G-protein-coupled P2Y12 subtype of the adenosine diphosphate (ADP) platelet receptor indefinitely and permanently [9]. Normally, ADP activates P2Y12, causing conformational changes in the surface molecule GPIIb/IIIa, which enhances GPIIb/IIIa affinity for the divalent bridging molecules fibrinogen and von Willebrand factor, allowing platelet aggregation [9]. P-glycoprotein affects the absorption and bioavailability of clopidogrel [10]. Platelet aggregation is decreased when Plavix is activated and blocks this G-protein-coupled receptor [9].

Aggrastat (Tirofiban) is a glycoprotein receptor antagonist, and it is administered intravenously [6]. It is a reversible, competitive inhibitor of the GPIIb/IIIa receptor, a platelet surface receptor involved in platelet aggregation [11]. It inhibits fibrinogen-dependent platelet aggregation and prolongs the bleeding time by preventing fibrinogen binding to the GPIIb/IIIa receptor [11,12].

After ingestion, passive diffusion rapidly absorbs aspirin in the GI tract. The absorption rate of aspirin depends on gastric potential hydrogen (pH) and dosage form. The plasma level of uncoated formulation peaks in 30-40 minutes, whereas enteric-coated formulation peaks in three to four hours [10,11]. The plasma half-life of aspirin is 15-20 minutes [8]. Tirofiban is administered intravenously. A rapid on-rate and off-rate characterize it, and it dissociates from the receptor at a half-life of 11 seconds [12]. The half-life of unbound tirofiban is 1.5-2 hours [12].

Aspirin is rapidly hydrolyzed into salicylic acid. Two major pathways eliminate salicylic acid by forming salicyluric acid and salicylphenolic glucuronide [13]. Fifteen percent of absorbed clopidogrel is metabolized in the liver by cytochrome P450 (CYP) enzymes to generate its active metabolite by bioactivation of clopidogrel via a two-step process. The other 85% is hydrolyzed to inactive carboxylic acid derivatives by carboxylesterase 1 (CES1) [10]. Tirofiban induces its effect in a dose- and concentration-dependent manner, and its effect on platelet aggregation depends on its plasma concentration [11].

The absorption of aspirin is first-order kinetic. Serum albumin binds aspirin and salicylic acid [14]. Salicylic acid is eliminated by renal excretion and metabolic conversion to conjugates with glycine and glucuronic acid [12]. The pharmacokinetics of clopidogrel and its primary inactive metabolites are linear [10]. The antithrombotic effect of clopidogrel is caused by the active metabolite produced by CYP P450 [15]. Tirofiban is excreted primarily through the kidneys and feces [11].

Because platelets cannot produce new COX, aspirin's antiplatelet effect lasts longer for the platelet's life (7-10 days) [16]. The active metabolite of clopidogrel interacts with the platelet's ADP P2Y12 receptor and permanently inhibits platelet aggregation, and its effect can last up to five to seven days [10]. ADP-induced platelet aggregation returns to baseline within four to eight hours of stopping tirofiban [12].

Efficacy of aspirin and Plavix for anticoagulation in cranial procedures

Cranial surgeries like craniotomies and craniectomies have been associated with an increased risk of thromboembolic events, including stroke and deep vein thrombosis. Anticoagulant medications like aspirin and Plavix are often used during the perioperative period to prevent these complications. In recent years, researchers have focused on the efficacy of these medications in lowering thromboembolic events in patients undergoing cranial surgery [17,18].

According to a thorough literature review, perioperative aspirin continuation in elective craniotomies is not associated with an increased risk of hemorrhagic complications [19]. Regarding thromboembolic events, our pooled study shows that aspirin continues to be beneficial; nevertheless, the result did not reach statistical significance [20].

Plavix, or clopidogrel, is a thienopyridine derivative that reduces thromboembolic events by suppressing platelet activation and aggregation [17,18]. Although unmeasured factors may limit intergroup comparability, patients on clopidogrel had a significantly higher incidence of severe traumatic cerebral hemorrhage. However, patients utilizing clopidogrel had a lower incidence of delayed traumatic cerebral hemorrhage after a negative initial cranial CT scan, according to a prospective cohort study [20].

Several studies have assessed the efficacy of aspirin and Plavix in preventing thromboembolic events in patients undergoing cranial surgery. According to one study, combining aspirin and Plavix effectively prevented thromboembolic events in patients undergoing craniotomies for cerebral aneurysms [21].

Aspirin and Plavix are often used together to maximize their antiplatelet effects. On the other hand, dual antiplatelet medicine has been related to an increased risk of bleeding difficulties. According to one study, using aspirin and Plavix together was associated with an increased risk of bleeding difficulties in individuals undergoing neuroendovascular surgery [18,21].

When deciding whether to use aspirin or Plavix for anticoagulation in cranial surgeries, it is critical to consider the patient's risk factors. Age, comorbidities, and concomitant medications should all be considered before making this decision [17].

Furthermore, the timing of aspirin or Plavix administration is essential to their efficacy. According to one study, the best results were obtained when aspirin and Plavix were administered at least 24 hours before the operation. Delayed aspirin or Plavix administration was associated with an increased risk of thromboembolic events [22].

Aspirin and Plavix are good antiplatelet medications that are often used to avoid thromboembolic events in patients undergoing cranial surgery. However, their use should be carefully considered on a case-by-case basis, considering each patient's risk factors and the time of administration. The risk of bleeding difficulties should be weighed against the potential benefits of anticoagulation [23].

Efficacy of Aggrastat for anticoagulation in cranial procedures

Aggrastat, a glycoprotein IIb/IIIa inhibitor, is increasingly used for anticoagulation in patients undergoing cranial surgery. Its efficacy in lowering thromboembolic events in these people has received much interest in recent years, and many studies have been conducted to look into it [24]. A meta-analysis of Tirofiban prophylactic treatment resulted in a significantly lower incidence of thromboembolic complications with no increase in hemorrhagic events or mortality compared to dual-antiplatelet therapy preventative treatment [25].

Aggrastat has a short half-life, which allows for fast reversal of its anticoagulant effects if necessary [24,26]. This property makes it an appealing option for patients undergoing cranial surgery, where the likelihood of bleeding complications is a concern. Furthermore, Aggrastat has been shown to have a lower risk of bleeding issues when compared to other anticoagulant medicines such as heparin [27].

According to one study, using Aggrastat significantly reduced postoperative thromboembolic events in people with craniotomies for cerebral aneurysms [24,28]. Similarly, another study found that Aggrastat helped people avoid thromboembolic events after carotid endarterectomy. These findings indicate Aggrastat's usefulness in preventing thromboembolic events in patients undergoing cranial surgery [29].

However, there are certain risks associated with using Aggrastat. According to the literature, Aggrastat administration has been linked to an increased risk of hypotension and bradycardia in patients undergoing carotid endarterectomy [26,28,29]. Another study found that using Aggrastat increased bleeding complications in those undergoing neuroendovascular surgery [29,30]. These findings highlight the need for carefully examining the individual patient's risk factors when deciding whether to use Aggrastat for anticoagulation in cranial surgeries [30].

According to one study, administering Aggrastat within 30 minutes after the start of the procedure resulted in optimal results [31]. Delayed Aggrastat administration, on the other hand, was associated with an increased incidence of thromboembolic events. Therefore, the timing of Aggrastat administration during procedures is important in maximizing its efficacy [31].

Therefore, compared to other anticoagulant medicines, using Aggrastat for anticoagulation in cranial surgeries seems beneficial in lowering thromboembolic events with a lower risk of bleeding difficulties. However, its use should be carefully considered on a case-by-case basis, as risk factors such as hypersensitivity to any Aggrastat components or acute pericarditis can be considered relative contraindications. More research is needed to determine the proper dose and duration of Aggrastat therapy in cranial operations [31,32].

Comparison of efficacy between aspirin, Plavix, and Aggrastat

Aspirin, Plavix, and Aggrastat have been used as anticoagulants in different cranial procedures, whereas aspirin and Plavix have been used for quite some time and Aggrastat has emerged as a new potential anticoagulant. There has been a debate regarding the best choice of anticoagulant drug for these procedures [33,34].

Various factors come into play when comparing all three drugs as suitable for cranial surgeries. Regarding reversibility, aspirin, and Plavix are irreversible anticoagulants, whereas Aggrastat works reversibly [33,34]. This factor is related to one of the major adverse effects of anticoagulant use: bleeding risk. In addition to that, Aggrastat has a short half-life compared to aspirin and Plavix [33,34]. Due to its shorter half-life and reversible mechanisms of action, Aggrastat significantly reduces bleeding complications after cranial surgeries, making it a safer option than aspirin and Plavix [33,34].

Moreover, Aggrastat has a much faster onset of action in minutes than aspirin and Plavix [33,34]. Related to this is the timing and dosage of the anticoagulant before the surgery [33,34]. The required timing to maximize the desired action and prevent the adverse effects was only 30 minutes before the procedure for Aggrastat, compared to 24 hours before the procedure required for aspirin and Plavix [33,34]. However, delayed administration of all three drugs was associated with an increased risk of thromboembolic effects [33,34].

On the other hand, when comparing treatment efficacy in preventing thromboembolic events in patients undergoing cranial surgeries, all three drugs have shown significant improvement. Aggrastat and Plavix effectively avoid thromboembolism in carotid endarterectomy patients [33,34].

Both monotherapy and a combination have also reduced overall mortality in post-stroke and acute aneurysmal patients. Aggrastat shows promise as an option to treat acute stroke patients for whom mechanical thrombectomies have failed and patients who have experienced a stroke outside the widow for thrombolytic therapy so long as there is no risk of symptomatic intracranial hemorrhage [33]. Some studies have shown increased hemorrhage rates in patients where antiplatelet therapy was initiated before or after exterior ventricular drain placement [34].

Furthermore, risk factors also play a crucial role in deciding the choice of anticoagulant for patients undergoing cranial procedures. Age, comorbidities, and concomitant medications all majorly affect the efficacy of aspirin and Plavix [33-35]. Aggrastat use is associated with bradycardia, hypotension, vasospasm, infarction, and increased bleeding complications in patients undergoing surgeries such as neuroendovascular operations, for which risk factor evaluation is critical before opting for the suitable choice of drugs [33-35].

However, even with infarction and hemorrhagic complications, patients pre-treated with an Aggrastat drip prior to neurological surgical interventions have lower rates of thromboembolic complications compared to oral dual-antiplatelet therapy, further highlighting the benefits of using Aggrastat as an alternative prophylactic antiplatelet pharmacological intervention [35]. Literature with smaller sample sizes has shown that its use in clinical practice would benefit patients in acute cases through mechanisms that would preserve blood flow and prevent irreversible ischemia when given within the allotted time parameters [33-36]. These patients have superior clinical outcomes, with high rates of neurological recanalization of vessels following neurological surgical interventions for acute cerebrovascular accidents and aneurysms and overall improved neurological function post-stroke due to stabilization of the inflamed stenotic lesions through platelet aggregation [36]. While more information is needed to understand the nuisances of the various pathologies that may impact the efficacy of Aggrastat use in clinical practice, the results have shown that more widespread randomized studies can collect more conclusive data [36]. Table 1 summarizes the key characteristics and features of aspirin, Plavix, and Aggrastat.

**Table 1:** Comparison of efficacy of antiplatelet medications in cranial procedures COX-1: cyclooxygenase 1; mg: milligrams, ADP-receptor: adenosine diphosphate receptor; mcg/kg: micrograms per kilogram

Medication	Mechanism of action	Dosage	Onset of action	Duration of action	Reversibility	Risk of bleeding	Efficacy in preventing thromboembolic events	Reference
Aspirin	Inhibits COX-1 enzyme	81-325 mg daily	1-2 hours	3-5 days	Irreversible	Low	Moderate	[33]
Plavix	Inhibits ADP- receptor on platelets	75 mg daily	2-4 hours	5-7 days	Irreversible	Moderate	High	[37]
Aggrastat	Inhibits glycoprotein IIb/IIIa receptor on platelets	Loading dose: 25 mcg/kg bolus, followed by 0.125 mcg/kg	Within minutes	4-6 hours	Reversible	Low	High	[38]

Speed and reversibility in the real-world

In cranial procedures, the speed of onset of antiplatelet medications gains significance in specific situations. For example, the management of intracranial aneurysms requires endovascular procedures such as stent-assisted coiling [35]. Major causes of morbidity and mortality after endovascular procedures include thrombosis and ischemic events [35]. Prophylactic use of antiplatelet therapy prior to stent-assisted coiling can minimize the number of thromboembolic events, thereby reducing morbidity and mortality [35]. The rapid onset of action of Aggrastat is advantageous since it will decrease the time needed to wait prior to starting the endovascular procedure [35].

Similarly, in acute ischemic stroke, prompt initiation of antiplatelet therapy is essential to restore blood flow to the brain and salvage ischemic tissue [8,9]. Aspirin irreversibly inhibits platelet aggregation and reduces the risk of recurrent ischemic events. While aspirin does not have a specific reversal agent, platelet transfusions can be considered in cases where immediate reversal is necessary. For example, if a patient on aspirin presents with an acute ischemic stroke and requires emergency thrombectomy, the availability of platelet transfusions can help manage bleeding risks during the procedure [9].

The ability to reverse the effects of antiplatelet medications quickly in cranial procedures can be limited due to various factors. The pharmacokinetics of the specific medications used in cranial procedures, such as aspirin or Aggrastat (tirofiban), play a role in their reversibility. For example, aspirin exerts an irreversible effect on platelet function, and its inhibitory effects persist for the lifespan of platelets until new platelets are generated [11,12]. This means that the effects of aspirin cannot be rapidly reversed with a specific antidote. Additionally, unlike certain anticoagulant medications, no widely available specific reversal agents are designed for antiplatelet medications. The decision to reverse antiplatelet medications in cranial procedures should be carefully considered, weighing the delicate balance between preventing thrombotic events and managing bleeding risks. Individual patient factors should be considered, including their clinical condition, bleeding risk, procedure urgency, and the potential consequences of interrupting or reversing the antiplatelet effects [17,20,25]. While the reversibility of antiplatelet medications may be limited in cranial procedures, other management strategies, such as hemostatic agents, endovascular interventions, or surgical techniques, can be employed to minimize bleeding risks and effectively manage complications [17].

Guide for antiplatelet medication selection in clinical situations

When selecting the best antiplatelet medication for a cranial procedure, physicians should consider the characteristics of aspirin, Plavix (clopidogrel), and Aggrastat (tirofiban) and the specific procedure involved: (i) assess the patient's thrombotic risk, considering their medical history and the indication for antiplatelet therapy, such as atrial fibrillation, recent coronary stent placement, or other high-risk conditions. Next, evaluate the urgency and nature of the cranial procedure. For instance, rapid bleeding control becomes paramount in an acute intracranial hemorrhage requiring emergency surgery. In elective cranial procedures, the balance between thrombotic and bleeding risks must be carefully considered. Examples of cranial procedures include craniotomy for tumor resection, aneurysm clipping, or arteriovenous malformation (AVM) surgery [19,20]. (ii) Assess the risk of thrombosis versus bleeding associated with the specific cranial procedure. For example, in an acute ischemic stroke requiring emergent thrombectomy, rapid restoration of blood flow is crucial, making the use of antiplatelet medications like aspirin important. On the other hand, in cranial surgeries with a high risk of bleeding, such as tumor resections or aneurysm repairs, the choice of antiplatelet medication and its reversibility become critical [20]. (iii) Engage in discussions with other providers, including neurosurgeons, neurologists, hematologists, and anesthesiologists, to tailor antiplatelet medication selection to the specific procedure. Collaborate with interventional neuroradiologists if endovascular procedures are considered, such as coil embolization or flow diverters for aneurysm treatment [19,25]. (iv) Select the most appropriate antiplatelet medication based on the above considerations. (v) Develop a perioperative antiplatelet management plan with the surgical team, considering the specific cranial procedure. This plan should include details on the timing of antiplatelet medication interruption, the need for bridging therapy if applicable, the initiation of post-procedure antiplatelet therapy, and monitoring parameters for bleeding and thrombotic risks. For instance, timing antiplatelet medication interruption and resumption in endovascular procedures is critical to balancing the risk of perioperative thrombosis and bleeding [19,20,25]. (vi) Monitor the patient's coagulation profile continuously and adjust the antiplatelet therapy as necessary, considering the desired target range, bleeding risks, and the specific characteristics of aspirin, Plavix, and Aggrastat in the context of the cranial procedure [20,25].

Dual antiplatelet therapy

Dual antiplatelet therapy (DAPT) involving aspirin and Plavix (clopidogrel) has been extensively studied and established as a standard regimen in cardiac and cerebrovascular conditions such as acute coronary syndrome and ischemic stroke. This combination enhances platelet inhibition by targeting different pathways involved in platelet activation [33]. However, the evidence specifically focusing on DAPT with aspirin and Plavix in cranial procedures is limited. The decision to use DAPT with aspirin and Plavix in cranial surgeries should be made on a case-by-case basis, considering a patient's clinical factors, bleeding risks, consultation with a multidisciplinary team of experts, and the specific procedure [34]. On the other hand, the role of Aggrastat in cranial procedures is not well established, and limited data are available on its use in this context. Careful consideration of the potential benefits of enhanced platelet inhibition against the increased bleeding risks associated with dual therapy is essential. Consulting recent research studies, clinical guidelines, and expert opinions from relevant medical societies and institutions can provide more specific and up-to-date information on the use of dual therapy with aspirin, Plavix, and Aggrastat in cranial procedures.

## Conclusions

Aspirin, Plavix, and Aggrastat have been used for anticoagulation in cranial procedures. These anticoagulants reduce the risk of stroke and DVT and are generally given before undergoing cranial surgery. Aspirin and Plavix together increase bleeding difficulties. Aggrastat is a new potential anticoagulant with a short half-life and a faster onset of action. It has the unique property of reversing its anticoagulant effect. It has significantly reduced bleeding issues and postoperative thrombotic events and decreased overall mortality in post-stroke and acute aneurysmal patients. We recommend using Aggrastat in cranial surgeries through this review though still more clinical trials are required for its implementation. Further investigation is warranted to evaluate how significant patients' risk factors are associated with the likelihood of severe adverse effects, as Aggrastat still has limited neuroendovascular research studies available.
